# Comparative metabolomics provides insights into the metabolic reprogramming of the predatory *Arma custos* (Fabricius) during low-temperature storage

**DOI:** 10.3389/finsc.2026.1820584

**Published:** 2026-06-05

**Authors:** Yifan Dong, Xiaoyan Yuan, Zihan Shi, Yansong Xiao, Shaolong Wu, Can Wang, Lin Tan, Weiai Zeng, Yusheng Wang

**Affiliations:** 1 Changsha Tobacco Company of Hunan Province, Changsha, China; 2 College of Plant Protection, Hunan Agricultural University, Changsha, China; 3 College of Horticulture, Hunan Agricultural University, Changsha, China; 4 Chenzhou Tobacco Company of Hunan Province, Chenzhou, China; 5 Tobacco Company of Hunan Province, Changsha, China

**Keywords:** *Arma custos*, carbohydrate metabolism, lipid metabolism, low-temperature stress, metabolomics

## Abstract

**Introduction:**

*Arma custos* (Fabricius) is a promising generalist predator capable of controlling a wide range of insect pests; however, a temporal mismatch between mass-rearing supply and pest outbreaks limits its commercial application. Although cold storage is commonly used to extend shelf life, it imposes substantial physiological stress that may compromise insect quality.

**Methods:**

To investigate the metabolic mechanisms underlying cold adaptation, we conducted comparative metabolomic analyses combined with enzyme activity assays on *A. custos* adults exposed to a gradual cooling protocol.

**Results:**

Despite a decline in survival during cold storage, the insects underwent extensive metabolic reprogramming in response to thermal stress. In total, 1,434 metabolites were identified, among which lipids and lipid-like molecules accounted for the largest proportion (43.72%). A total of 1,275 differentially expressed metabolites (DEMs) were detected, indicating pronounced metabolic reorganization and overall downregulation during prolonged cold exposure. KEGG pathway enrichment analysis identified eight major metabolic pathways and 46 key DEMs, suggesting that lipid and carbohydrate metabolism are central to cold tolerance. Mechanistically, the accumulation of long-chain polyunsaturated phospholipids and sphingomyelin is hypothesized to represent a crucial strategy for maintaining membrane fluidity, whereas the downregulation of ceramide may contribute to the suppression of apoptosis. In addition, elevated trehalase activity and the accumulation of compatible solutes likely function as essential cryoprotectants and antioxidants.

**Discussion:**

Collectively, these results provide a systematic characterization of the metabolic landscape of *A. custos* under low-temperature stress and offer a theoretical foundation for future functional studies and for optimizing cold storage strategies to enhance the performance of this biocontrol agent.

## 1 Introduction

*Arma custos* (Fabricius) (Hemiptera: Pentatomidae), synonymous with *Arma chinensis* (Fallou) (1), is an important generalist predator widely distributed across East Asia (2–4). It effectively suppresses a broad range of agricultural and forestry pests within the orders Lepidoptera, Coleoptera, and Hemiptera (5, 6), including *Spodoptera frugiperda* (7), *Plutella xylostella* (8), and *Leptinotarsa decemlineata* (9). Owing to its high predatory efficiency, *A. custos* is mass-reared and commercially deployed for pest control in tobacco, vegetables, and ornamental landscapes (10, 11), playing a key role in sustainable pest management programs. However, the practical use of mass-reared *A. custos* is frequently limited by temporal asynchrony between predator availability and pest outbreaks, resulting in reduced control efficacy and increased maintenance costs (12). Therefore, the development of effective storage techniques to extend the shelf-life of *A. custos* is essential to mitigate this mismatch.

Cold storage is widely applied to prolong the shelf-life of natural enemies (13), including predatory insects, and to ensure a stable supply for biological control programs (14). As ectothermic organisms, insects are highly sensitive to the suboptimal temperatures associated with cold storage, which often impose fitness costs affecting growth, reproduction, and survival (15, 16). For example, low-temperature treatment significantly reduced survival in *Trichogramma brassicae* (17) and *Orius similis* (18). Under cold stress, insects adopt a range of species-specific physiological strategies to enhance survival, primarily involving metabolic reorganization, membrane remodeling, and the accumulation of cryoprotectants and antioxidants. *Drosophila melanogaster* and *Cinara tujafilina* maintain membrane fluidity by increasing the proportion of phosphatidylethanolamine or unsaturated fatty acids, accompanied by the accumulation of osmoprotectants such as proline and trehalose (19, 20). In addition, *C. tujafilina* exhibits pronounced metabolic adjustments, including enhanced glycolysis and suppression of the tricarboxylic acid (TCA) cycle (20). Similarly, predatory insects such as *Adalia decempunctata* and *Hippodamia variegata* improve cold hardiness through the accumulation of diverse cryoprotectants (e.g., glucose and glycerol) and the activation of antioxidant enzymes (21, 22). Collectively, these physiological and biochemical modifications constitute the fundamental mechanistic basis of cold resistance and survival.

Low-temperature treatments have also been investigated in *A. custos*. Xia et al. (16) evaluated its development and survival under various constant temperatures. Li (23) identified 4 °C as the optimal storage temperature for adults, whereas eggs and nymphs experienced 100% mortality within seven days under the same conditions. Adult cold tolerance was found to depend on nutritional status and was associated with marked alterations in low-molecular-weight carbohydrates, glycerol, fatty acids, and enzyme activities. Likewise, Guo et al. (12) reported high survival rates in adults stored at 5 °C, accompanied by the accumulation of energy reserves and cryoprotectants, as well as significantly elevated antioxidant enzyme activities. Cold acclimation has increasingly been recognized as a critical strategy for enhancing storage efficiency and cold tolerance in natural enemies (24). Beyond extending shelf-life and improving survival across diverse taxa, acclimation can mitigate the sublethal effects of cold storage on insect performance (25–27). These benefits are supported by a complex array of physiological adjustments induced during acclimation (28, 29). Although a cold storage protocol incorporating acclimation has been explored in *A. custos* (30), the metabolic landscape underlying cold acclimation remains unclear. In the present study, comparative metabolomics combined with enzyme activity assays was employed to characterize physiological changes in adult *A. custos* during cold storage and to identify potential key metabolic determinants and regulatory pathways involved in cold adaptation. These findings provide important insights for optimizing low-temperature storage protocols for *A. custos* and contribute to a broader understanding of environmental adaptation strategies in predatory natural enemies.

## 2 Materials and methods

### 2.1 Insect rearing and cold-acclimation treatments

The *A. custos* individuals used in this study originated from a stable colony maintained for more than 30 generations at the College of Plant Protection, Hunan Agricultural University. Insects were fed fresh *Antheraea pernyi* pupae and reared under controlled environmental conditions of 26 ± 1 °C, 70 ± 5% RH, and a 16 L: 8D photoperiod.

**Figure 1:**
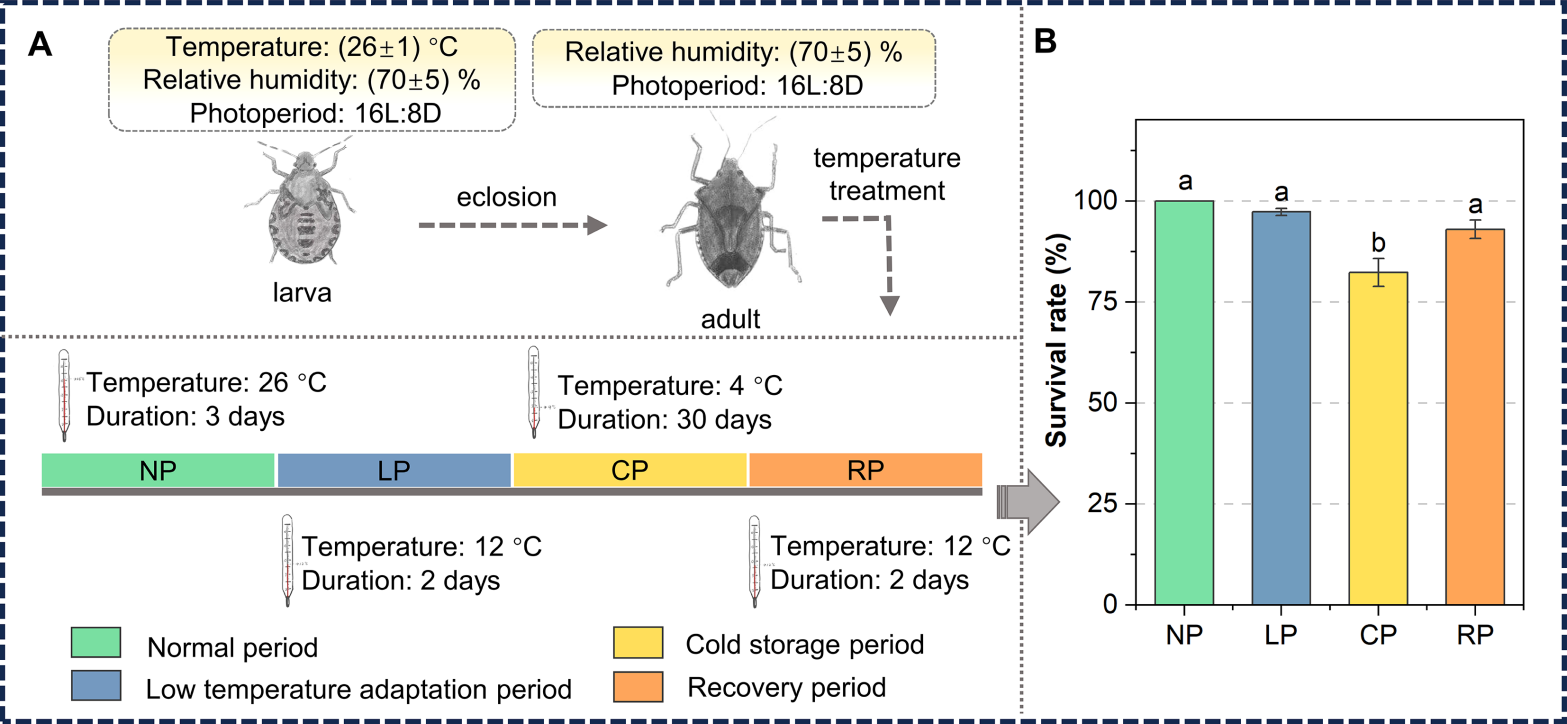
Experimental design flowchart **(A)** and survival rate of *Arma custos* during cold storage under a gradually decreasing temperature treatment **(B)**.

Given that *A. custos* adults exhibit greater cold tolerance than eggs and nymphs and are the primary stage released in biological control programs (23), they were prioritized for this study. A gradual temperature reduction regime was applied to facilitate cold storage of *A. custos*, following previously reported protocols (**Figure 1A**) (30). Healthy 3-day-old adults with uniform body size and no visible morphological deformities were selected for subsequent experiments (designated as the normal period, NP). One hundred well-fed adults (male: female = 1:1) were then placed in a plastic box (30 cm × 20 cm × 10 cm) lined with filter paper and maintained at 12 °C in a low-temperature incubator (designated as the low-temperature adaptation period, LP). After two days of acclimation, the insects were transferred to 4 °C for cold storage and maintained under this condition for 30 days (designated as the cold storage period, CP). Following cold storage, the insects were returned to 12 °C for two days to readjust to normal environmental conditions (designated as the recovery period, RP). To mimic natural storage environments, the adults were permitted to mate without restriction throughout the experiment.

Throughout the cold storage process, all *A. custos* individuals were provided with sufficient water but no food or illumination. Survival rates across the four stages were assessed using approximately 100 insects per group at each stage, with three biological replicates. Significant differences among groups were evaluated using a one-way ANOVA followed by Tukey’s HSD test (*p* < 0.05).

### 2.2 Metabolite extraction

Insect samples were collected at each designated time point for metabolomic and trehalase activity analyses. Under a laminar flow hood, intact insects were briefly rinsed with 1 × PBS, gently blotted dry with sterile filter paper, immediately snap-frozen in liquid nitrogen, and stored at -80 °C until use. Each treatment included six biological replicates, with each replicate consisting of two female and two male adults.

Adult *A. custos* samples were ground into fine powder under liquid nitrogen. A 50 mg aliquot of each constant-weight sample was then mixed with 800 μL of methanol/water (4:1, v/v) extraction solvent containing the internal standard L-2-chlorophenylalanine (final concentration 0.02 mg/mL). The mixtures were homogenized at 50 Hz for 6min at -20 °C using a high-throughput tissue grinder.

Following homogenization, samples were subjected to ultrasonic extraction at 5 °C and 40 kHz for 30 min. The extracts were then incubated at -20 °C for 30 min and centrifuged at 13,000 r/min for 15 min at 4 °C. The resulting supernatants were carefully collected and transferred into sample vials for normalized metabolite analysis using the UPLC-MS/MS system (31).

### 2.3 Metabolomic determination

Metabolite separation and detection were performed using an UHPLC-Q Exactive HF-X system equipped with an ACQUITY HSS T3 column (Waters, Milford, USA) and an electrospray ionization (ESI) source. The mobile phase consisted of phase A (95% water and 5% acetonitrile containing 0.1% formic acid) and phase B (47.5% acetonitrile, 47.5% isopropanol, and 5% water containing 0.1% formic acid). Chromatographic conditions included a flow rate of 0.40 mL/min, a column temperature of 40 °C, and a 5 μL injection volume. The gradient elution program was set as follows:: 0–3.5 min, 100–75.5% A (0–24.5% B) at 0.4 mL/min; 3.5–5 min, 75.5–35% A (24.5–65% B) at 0.4 mL/min; 5–5.5 min, 35–0% A (65–100% B) at 0.4 mL/min; 5.5–7.4 min, 0% A (100% B) at 0.6 mL/min; 7.4–7.6 min, 0–48.5% A (100–51.5% B) at 0.6 mL/min; 7.6–7.8 min, 48.5–100% A (51.5–0% B) at 0.5 mL/min; 7.8–10 min, 100% A (0% B) at 0.4 mL/min, for column equilibration.

The ion source temperature and capillary temperature were set to 425 °C and 325 °C, respectively. The sheath gas and auxiliary gas flow rates were maintained at 50 and 13 arb, respectively. The normalized collision energy for MS/MS was set to 20–40–60 V in rolling mode. The spray voltage was set to (+) 3,500 V and (−) 3,500 V. The resolution was 60,000 for full MS scans and 7,500 for MS/MS scans, with a mass scan range of m/z 70–1,050. Data acquisition was conducted in both positive and negative ion modes to comprehensively capture metabolite signals.

To ensure analytical stability and reproducibility, pooled quality control (QC) samples were prepared by combining equal aliquots from each sample extract. QC samples were injected at regular intervals throughout the analytical run to monitor system performance.

### 2.4 Metabolomics analysis

To validate the stability and reproducibility of the UPLC-MS/MS analysis, the total ion chromatograms (TICs) of the QC samples were superimposed and examined. Raw chromatographic and mass spectrometric data were processed using Progenesis QI 2.3 software (Nonlinear Dynamics, Waters, USA), including peak detection and alignment to generate a data matrix comprising retention time (RT), mass-to-charge ratio (m/z), and peak area for each sample. To address redundancy between positive (ESI+) and negative (ESI−) ionization modes, metabolites detected in both modes were represented only by the feature with the higher MS/MS spectral matching score. During data preprocessing, only metabolic features detected in at least 80% of all samples were retained. After normalization by total ion intensity (summation), metabolites with a relative standard deviation (RSD) greater than 30% in QC samples were excluded from further analysis. The resulting data matrix was log10-transformed and uploaded to the Majorbio cloud platform (https://cloud.majorbio.com) for subsequent analyses.

Metabolite annotation was performed using the Human Metabolome Database (HMDB) and the Kyoto Encyclopedia of Genes and Genomes (KEGG) databases. Significant differences among experimental groups were evaluated using orthogonal partial least squares discriminant analysis (OPLS-DA) implemented in the ropls 1.6.2 R package. Variable importance in projection (VIP) values were calculated using MetaboAnalyst 4.0 and the ropls 1.6.2 R package. In accordance with a previous study, differentially expressed metabolites (DEMs) were rigorously identified using a combination of multidimensional criteria: VIP > 1, a raw *p*-value < 0.05, and |log_2_foldchange| > 1 (32). These DEMs were subsequently subjected to KEGG pathway enrichment analysis to determine significantly affected metabolic pathways.

K-means clustering analysis was conducted using the kmeans function, with the optimal number of clusters determined by the cascadeKM function in the vegan 2.6–2 R package (11). Differences between groups of metabolites involved in lipid and carbohydrate metabolism were assessed using *t*-tests to determine statistical significance.

### 2.5 Determination of trehalase activity

Since trehalose metabolism is a highly dynamic process, steady-state levels alone are inadequate to fully capture carbohydrate mobilization and energy flux under stress (33). Consequently, we further assayed trehalase activity to monitor metabolic turnover in these samples. To assess changes in trehalase activity in *A. custos* during cold storage, enzyme activity was measured in each treatment group using a Trehalase Assay Kit (Nanjing Jiancheng, A150-1-1, China) according to the manufacturer’s instructions (34). A 100 mg sample, cryogenically ground in liquid nitrogen, was used for each assay.

Absorbance (A) was recorded at 505 nm using a microplate reader (BioTek, Vermont, USA). Trehalase activity was calculated using the following formula: trehalase activity (U/g) = 366 × (ΔA+0.0042)/weight.

Trehalase activity data were also analyzed by one-way ANOVA followed by Tukey’s HSD test to evaluate significant differences among groups (*p* < 0.05).

## 3 Results

### 3.1 Survivorship and overview of metabolomic profiles

Newly emerged adults of *A. custos* were subjected to a sequential temperature regimen consisting of a normal period (NP, three days at 26 °C), a low-temperature adaptation period (LP, two days at 12 °C), a cold storage period (CP, 30 days at 4 °C), and a recovery period (RP, two days at 12 °C). The survival rate of *A. custos* was recorded at each time point during the gradually decreasing temperature treatment. Significant differences in survival were observed among stages (*F* = 13.476; *df* = 3,8; *p* = 0.002). Notably, the cold storage period exhibited a significantly lower survival rate (82.29%) than the other stages (> 93.00%) (**Figure 1B**).

**Figure 2:**
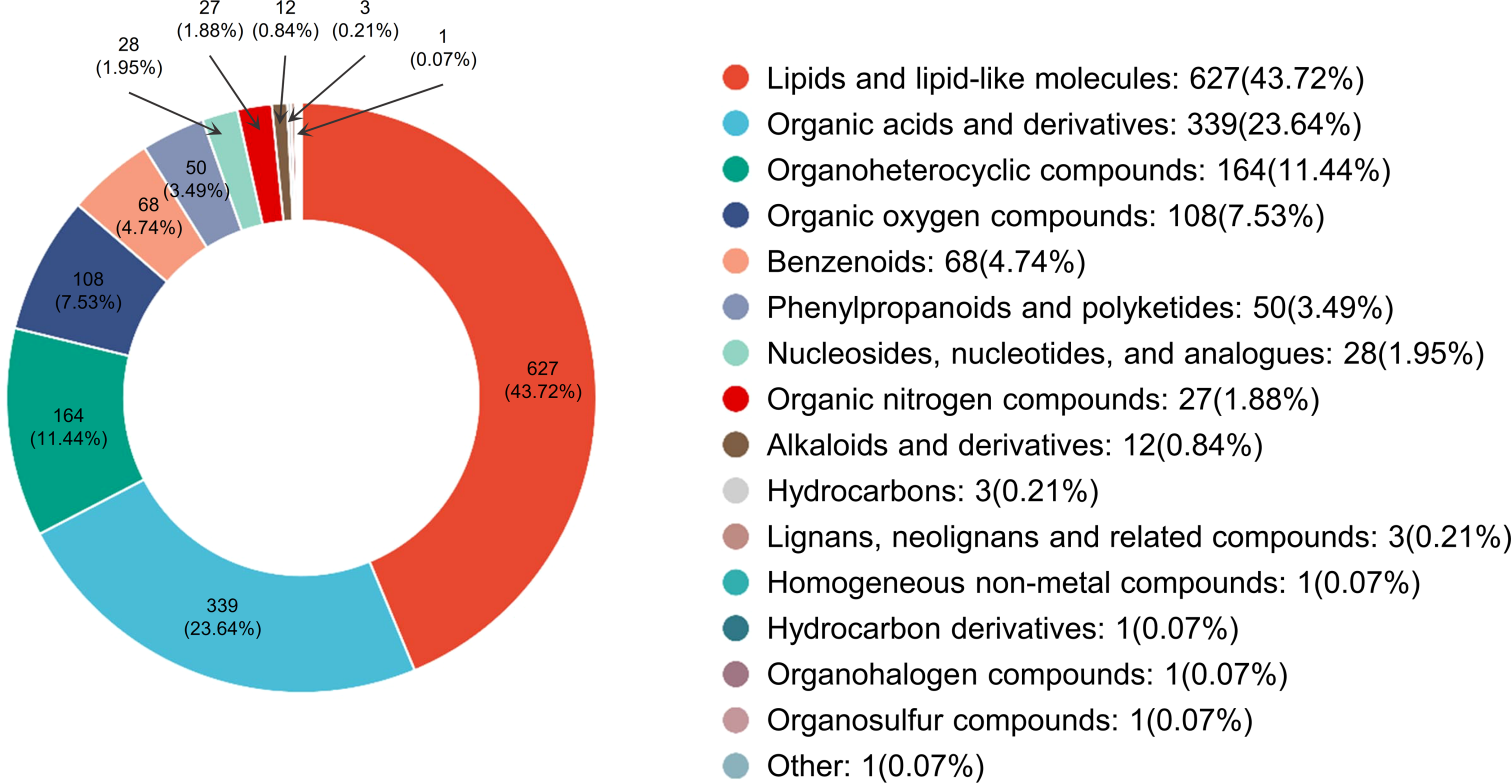
HMDB-based classification of identified metabolites.

To characterize metabolic responses, the metabolomic profiles of *A. custos* were analyzed using UPLC-MS/MS with relative quantification. The TICs of the pooled QC samples exhibited a high degree of overlap, demonstrating excellent signal stability and instrument reliability throughout the experiment (**Supplementary Figure 1**). A total of 16,923 ion peaks were detected, corresponding to 1,434 identified metabolites. Based on HMDB classification, these metabolites were assigned to 16 categories (**Figure 2**). Lipids and lipid-like molecules constituted the largest proportion (627, 43.72%), followed by organic acids and derivatives (339, 23.64%), organoheterocyclic compounds (164, 11.44%), and organic oxygen compounds (108, 7.53%). The coordinated variation of lipids, organic acids, organoheterocyclic compounds, and organic oxygen compounds may collectively form a “cold-tolerance metabolic module” that supports cellular homeostasis in *A. custos*. The remaining metabolites were mainly classified as benzenoids (68, 4.74%), phenylpropanoids and polyketides (50, 3.49%), nucleosides, nucleotides, and analogues (28, 1.95%), and organic nitrogen compounds (27, 1.88%), among others.

**Figure 3:**
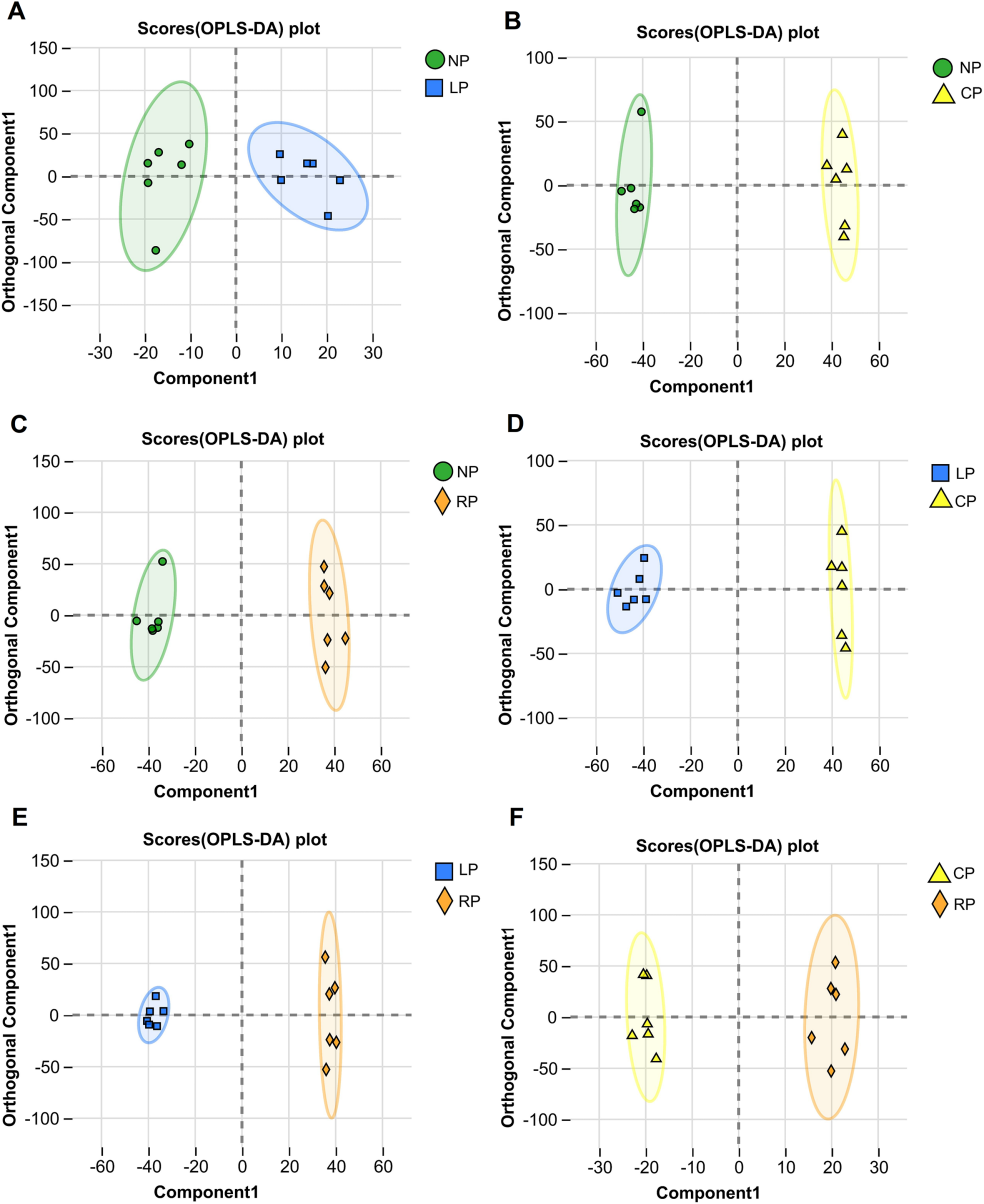
OPLS-DA score plots for pairwise comparisons: LP vs. NP **(A)**, CP vs. NP **(B)**, RP vs. NP **(C)**, CP vs. LP **(D)**, RP vs. LP **(E)**, and RP vs. CP **(F)**. NP, normal period; LP, low temperature adaptation period; CP, cold storage period; RP: recovery period.

To explore global metabolic differences among the NP, LP, CP, and RP groups and to evaluate within-group variation, OPLS-DA was conducted for pairwise comparisons. The OPLS-DA score plots demonstrated clear separation between the groups in all comparisons (LP vs. NP, CP vs. NP, RP vs. NP, CP vs. LP, RP vs. LP, and RP vs. CP), with samples from each group tightly clustered within the 95% Hotelling's T2 confidence ellipse (**Figure 3**). These results indicate pronounced metabolic divergence among treatments and high reproducibility among biological replicates, confirming that cold storage markedly reshapes the metabolic profile.

### 3.2 Identification of differentially expressed metabolites

Volcano plots were generated to visualize the overall patterns of differentially expressed metabolites in *A. custos* during cold storage. In total, 1,275 DEMs were identified based on the thresholds VIP > 1, *p* < 0.05, and |log_2_FC| > 1. According to HMDB classification, these DEMs were distributed across 13 categories, with the predominant classes consistent with the overall metabolite distribution described above (**Supplementary Figure 2**). 

**Figure 4:**
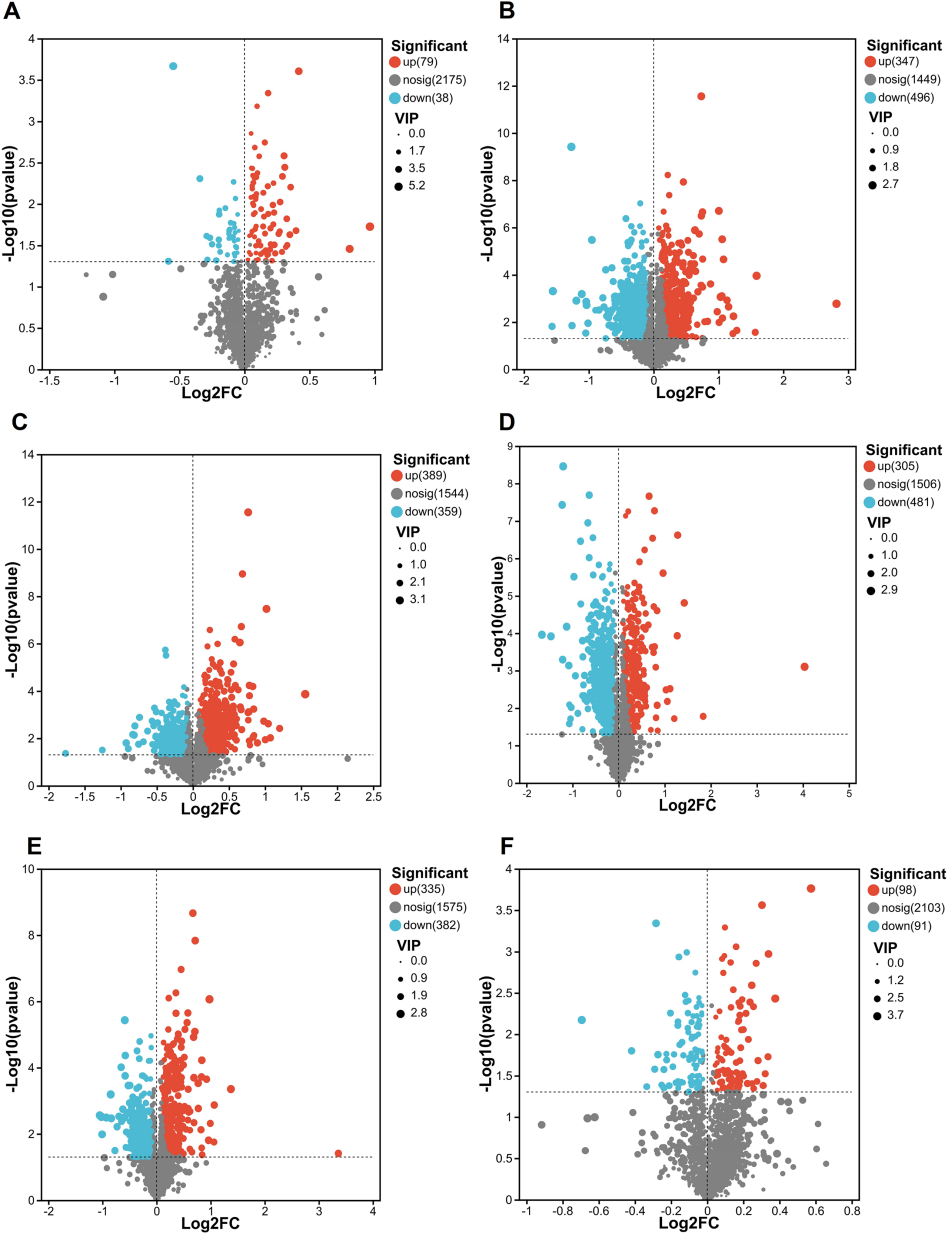
Volcano plots of pairwise comparisons: LP vs. NP **(A)**, CP vs. NP **(B)**, RP vs. NP **(C)**, CP vs. LP **(D)**, RP vs. LP **(E)**, and RP vs. CP **(F)**. NP, normal period; LP, low temperature adaptation period; CP, cold storage period; RP, recovery period. DEMs were rigorously defined by the criteria: VIP > 1, a raw *p*-value < 0.05, and |log_2_foldchange| > 1.

Specifically, 117 DEMs were identified in LP *v*s. NP (79 up-regulated and 38 down-regulated) (**Figure 4A**). In CP *vs*. NP, 843 DEMs were detected (347 up-regulated and 496 down-regulated) (**Figure 4B**), whereas 748 DEMs were identified in RP *vs*. NP (389 up-regulated and 359 down-regulated) (**Figure 4C**). In comparisons relative to LP, 786 DEMs were observed in CP *vs*. LP (305 up-regulated and 481 down-regulated) (**Figure 4D**), and 717 DEMs were detected in RP *vs*. LP (335 up-regulated and 382 down-regulated) (**Figure 4E**). Finally, 189 DEMs were identified in RP *vs*. CP (98 up-regulated and 91 down-regulated) (**Figure 4F**).

Notably, the number of DEMs was markedly lower in LP *vs*. NP and RP *vs*. CP compared with the other comparisons. These findings indicate that cold storage, particularly prolonged exposure during the CP stage, induces substantial metabolic reprogramming in *A. custos*, whereas moderate-temperature acclimation during LP and RP exerts comparatively modest effects on the metabolome.

### 3.3 Clustering analysis of differentially expressed metabolites

**Figure 5:**
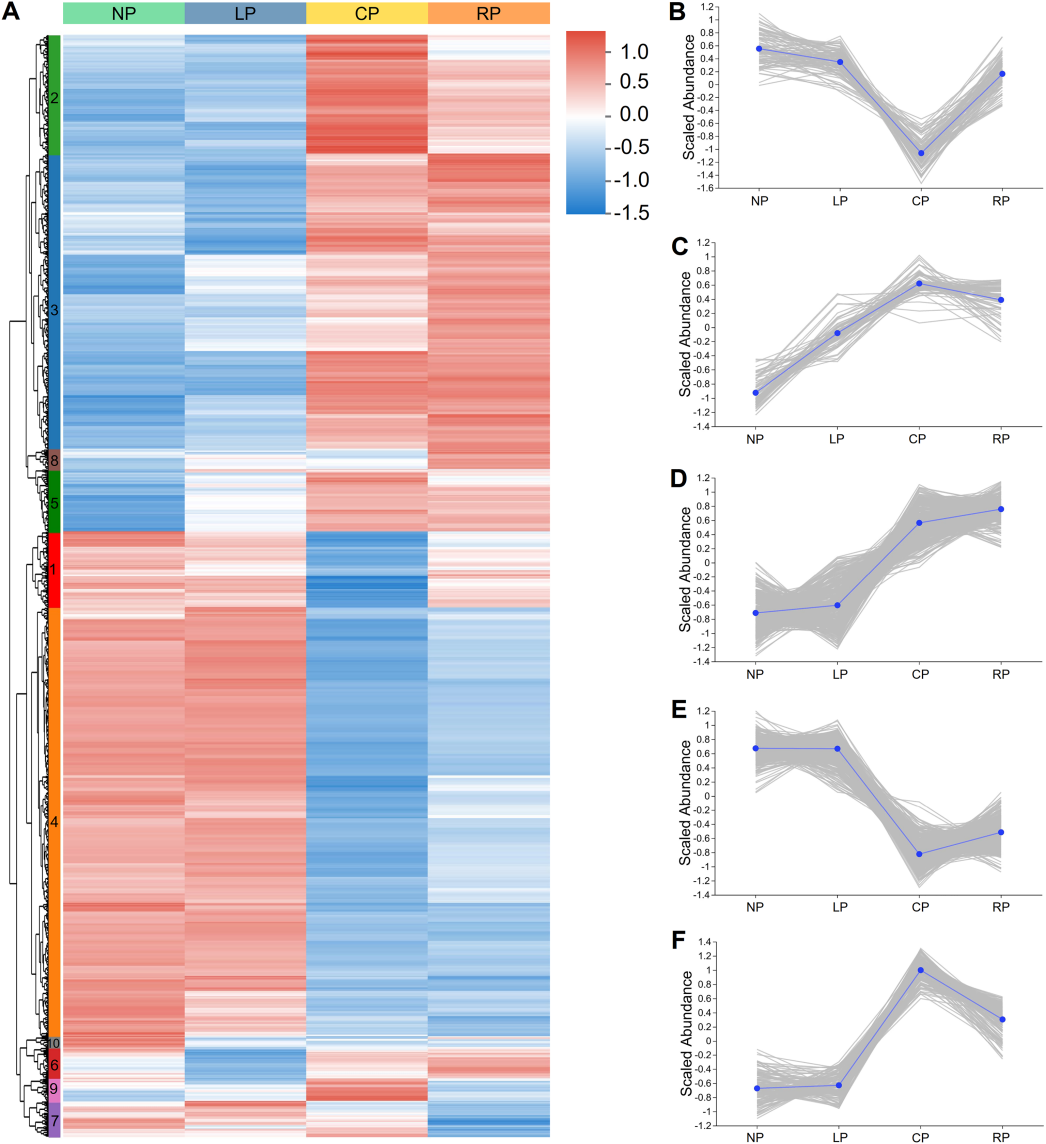
Clustering analysis of differentially expressed metabolites during cold storage. **(A)** K-means clustering analysis; **(B–F)** Representative trend profiles of DEMs with consistent expression patterns: cluster 1 **(A)**, cluster 2 **(B)**, cluster 3 **(C)**, cluster 4 **(D)**, cluster 5 **(F)**. The color scale indicates relative abundance, ranging from -1.5 to 1.0. Different colors in the top bar represent different stages, and the dendrogram on the left indicates cluster classification. NP, normal period; LP, low temperature adaptation period; CP, cold storage period; RP, recovery period.

Hierarchical clustering analysis based on scaled abundance was conducted to characterize metabolic shifts in *A. custos* throughout cold storage. To further delineate global expression trends, k-means clustering was performed on all identified DEMs. The analysis revealed distinct stage-specific expression patterns among the NP, LP, CP, and RP groups, identifying metabolites uniquely up- or down-regulated at particular stages and partitioning the 1,275 DEMs into 10 clusters (**Figure 5A**).

The five largest clusters, comprising 1,137 metabolites, displayed clear and consistent temporal patterns and were therefore selected for detailed analysis. Their dynamic trends are illustrated in **Figure 5B-F** as trend profiles. Clusters 1 and 4, containing 585 metabolites, exhibited similar expression trajectories, with high abundance during the early stages (NP and LP) followed by a marked decline at the CP stage (**Figures 5B, E**). Notably, metabolites in cluster 1 showed partial recovery during the RP stage. In contrast, clusters 2, 3, and 5, comprising 552 metabolites, generally accumulated during the later stages (**Figures 5C, D, F**). Among these, clusters 2 and 5 reached peak abundance at the CP stage, whereas cluster 3 displayed a continuous upward trend across stages.

Functional classification revealed that cluster 1 was predominantly enriched in lipids and lipid-like molecules, including fatty acyls, glycerophospholipids, sphingolipids, steroids, and their derivatives. Cluster 2 comprised lipid-related compounds, amino acid derivatives, and carbohydrate-associated metabolites. Cluster 3 was mainly characterized by amino acids, peptides, and other nitrogen-containing metabolites. Cluster 4 included signaling lipids, membrane lipid metabolites, and carbohydrate-related compounds. Cluster 5 consisted primarily of phospholipids, nucleotide-related metabolites, carbohydrate-associated metabolites, and other small molecules involved in biosynthetic processes.

### 3.4 Enrichment analysis of differentially expressed metabolites

**Figure 6:**
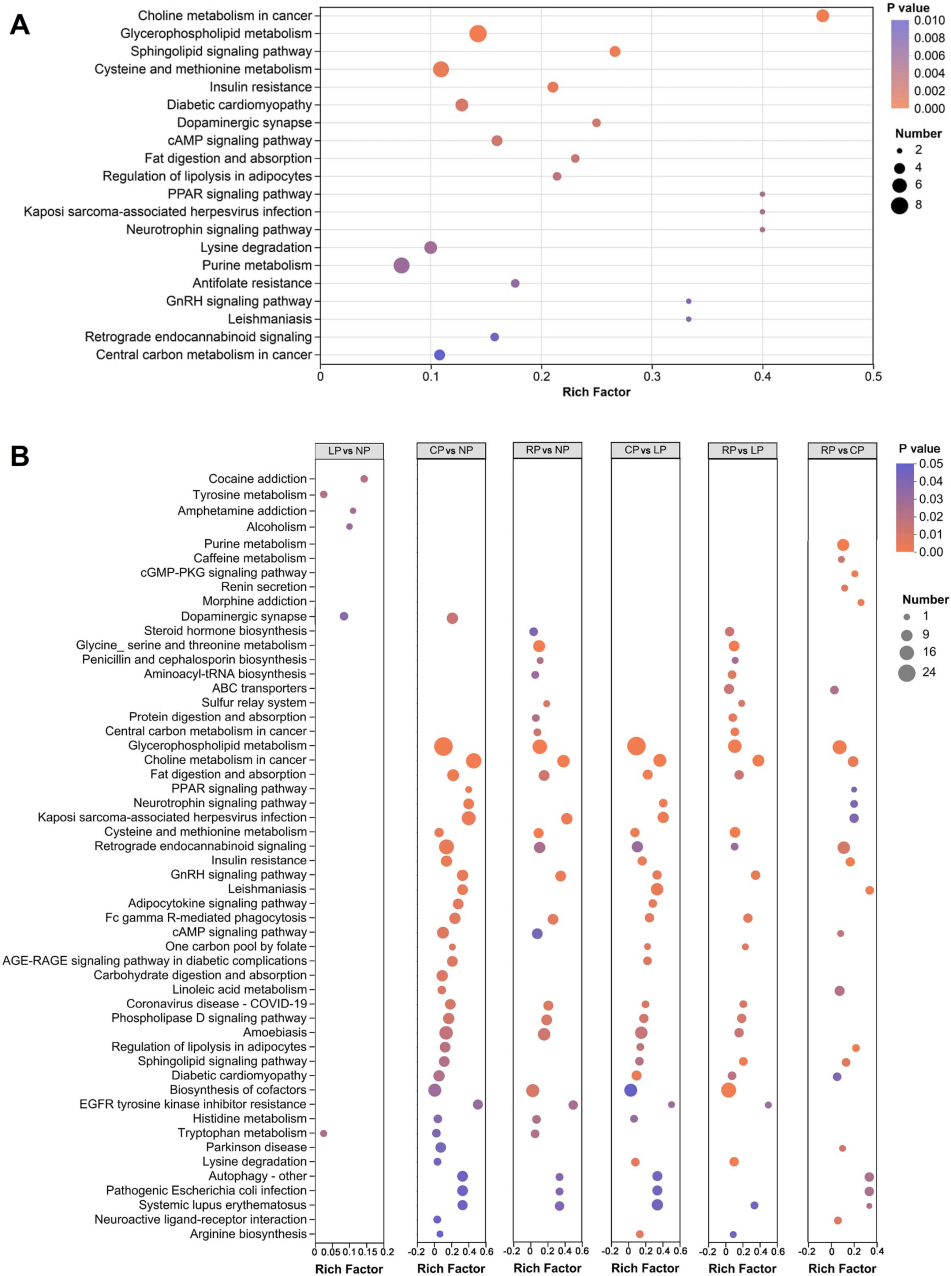
KEGG enrichment analysis of differentially expressed metabolites during cold storage. **(A)** KEGG enrichment of 1,137 DEMs from the top five representative clusters; **(B)** KEGG enrichment of DEMs across pairwise comparison groups. Dot size represents the number of DEMs associated with each pathway, and dot color indicates the *p*-value. NP, normal period; LP, low temperature adaptation period; CP, cold storage period; RP, recovery period.

To gain insights into the molecular mechanisms underlying cold storage adaptation in *A. custos*, functional annotation and enrichment analyses were performed on the 1,137 DEMs from the five major clusters. These metabolites were mapped to 173 metabolic pathways, primarily associated with lipid metabolism (14.59%), amino acid metabolism (12.43%), cancer: overview (8.11%), nervous system (6.76%), digestive system (6.22%), and endocrine system (5.68%) (**Supplementary Table 1**). Among these, lipid metabolism-related pathways—including choline metabolism in cancer, glycerophospholipid metabolism, and the sphingolipid signaling pathway—showed particularly high enrichment factors (**Figure 6A**). In addition, carbohydrate metabolism-associated pathways, such as insulin resistance, the cAMP signaling pathway, and central carbon metabolism in cancer, were significantly enriched.

KEGG enrichment analysis was further conducted for the six pairwise comparisons (**Figure 6B**). For each comparison, the top 20 metabolic pathways were selected based on *p*-values, rich factors, and the number of associated metabolites. Lipid-related pathways—particularly glycerophospholipid metabolism, choline metabolism in cancer, and retrograde endocannabinoid signaling—were consistently enriched in five comparisons (CP *vs*. NP, RP *vs*. NP, CP *vs*. LP, RP *vs*. LP, and RP *vs*. CP). Notably, glycerophospholipid metabolism and choline metabolism in cancer involved a large number of DEMs, especially in the CP *vs*. NP and CP *vs*. LP comparisons. Additional significantly enriched pathways included fat digestion and absorption, cysteine and methionine metabolism, and Fc gamma R-mediated phagocytosis. Among all comparisons, CP *vs*. NP exhibited the highest number of significantly enriched pathways, followed sequentially by CP *vs*. LP, RP *vs*. LP, RP *vs*. NP, and RP *vs*. CP, whereas LP *vs*. NP showed the fewest enriched pathways.

### 3.5 Lipid and carbohydrate metabolism affected by low-temperature

**Figure 7:**
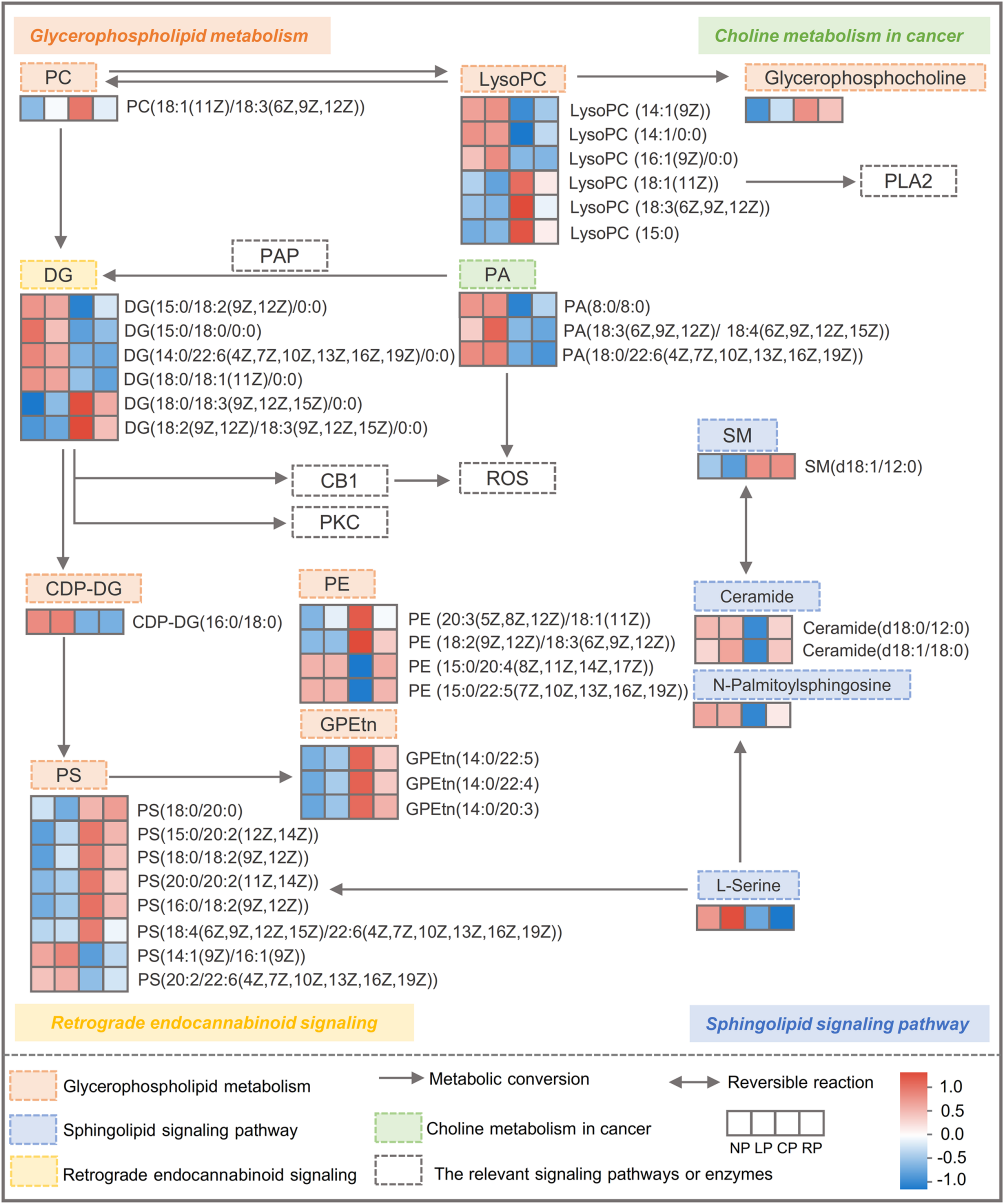
Visualization of the metabolic network formed by differentially expressed metabolites involved in lipid metabolism pathways. Color intensity represents the normalized abundance of each compound. NP, normal period; LP, low temperature adaptation period; CP, cold storage period; RP, recovery period.

**Figure 8:**
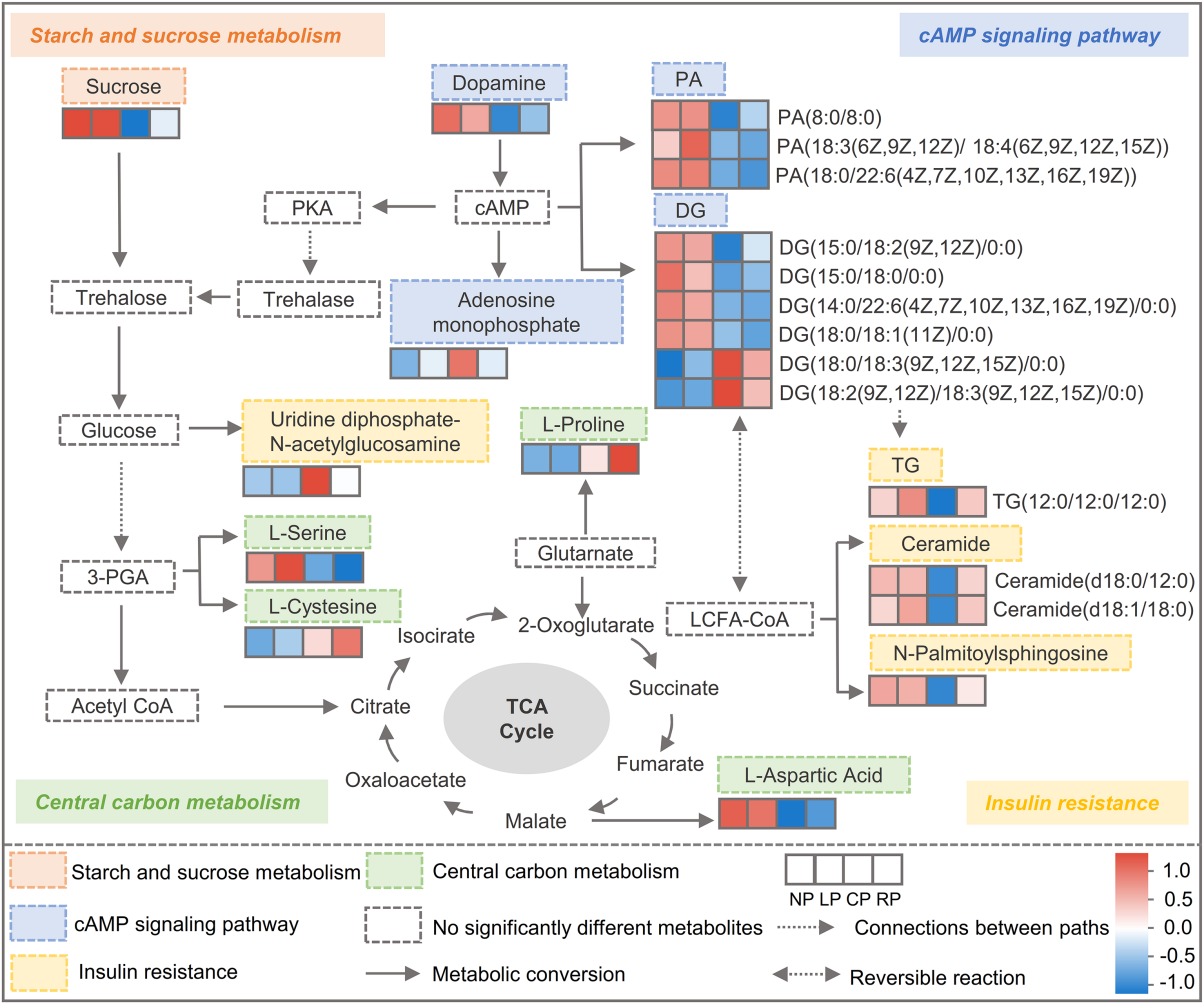
Visualization of the metabolic network formed by differentially expressed metabolites involved in carbohydrate metabolism pathways. Color intensity represents the normalized abundance of each compound. NP, normal period; LP, low temperature adaptation period; CP, cold storage period; RP, recovery period.

Integrated analysis of KEGG pathway enrichment and biological function indicated that lipid metabolism pathways (e.g., glycerophospholipid metabolism, sphingolipid signaling pathway, choline metabolism in cancer, and retrograde endocannabinoid signaling pathway) and carbohydrate metabolism pathways (e.g., starch and sucrose metabolism, central carbon metabolism, cAMP signaling pathway, and insulin resistance pathway) were significantly altered during cold storage of *A. custos*. To further explore the underlying mechanisms, network pathway maps were constructed to illustrate the specific roles of these metabolic processes in cold adaptation (**Figure 7**, **8**).

Across the four lipid-related pathways, 38 DEMs were identified, including 20 up-regulated and 18 down-regulated metabolites during cold storage (**Figure 7**; **Supplementary Figure 3**). Glycerophosphocholine (GPC), glycerophosphatidylethanolamine (GPEtn), phosphatidylcholine (PC), phosphatidylserine (PS), and sphingomyelin (SM) generally exhibited increasing trends. In contrast, cytidine diphosphate diacylglycerol (CDP-DAG), diacylglycerol (DG), L-serine, N-palmitoylsphingosine, and phosphatidic acid (PA) showed overall declines. Ceramide displayed a decreasing trend followed by a subsequent increase. Meanwhile, lysophosphatidylcholine (LysoPC) and phosphatidylethanolamine (PE) did not exhibit consistent patterns of change.

**Figure 9:**
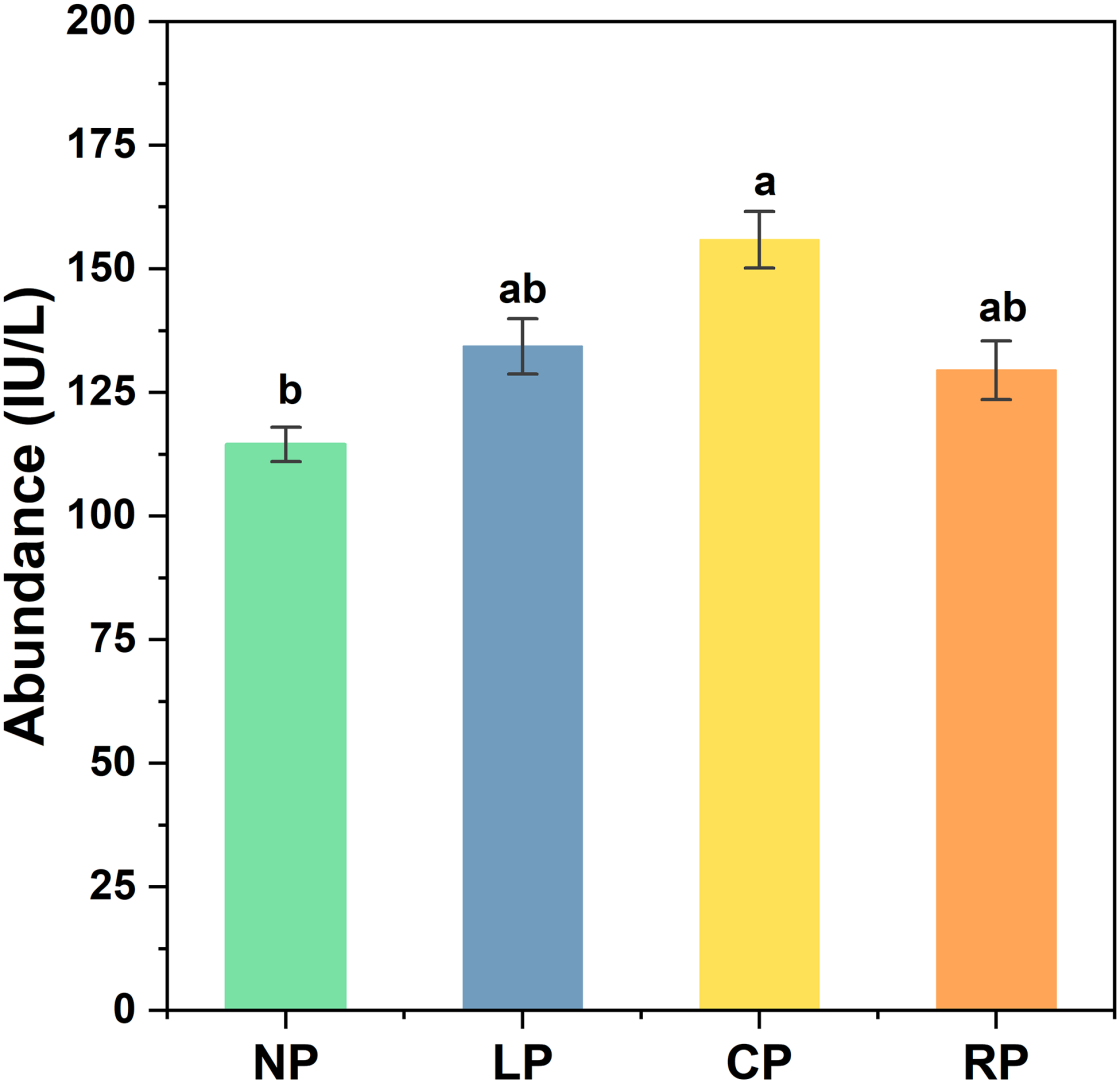
Dynamic changes in trehalase activity during cold storage. Different letters above the bars indicate significant differences (ANOVA, *p* < 0.05). NP, normal period; LP, low temperature adaptation period; CP, cold storage period; RP, recovery period.

Within the four carbohydrate-related pathways, 21 DEMs were identified, including 6 up-regulated and 15 down-regulated metabolites (**Figure 8**; **Supplementary Figure 3**). Most differential carbohydrate metabolites showed a general decline during cold storage, with key metabolites such as dopamine, L-aspartic acid, and sucrose exhibiting highly significant reductions after the cold storage period (CP). In addition, trehalase activity displayed an initial increase followed by a decrease during cold storage, indicating substantial perturbation of associated metabolic processes (*F* = 3.584; *df* = 3, 12; *p* = 0.047) (**Figure 9**).

## 4 Discussion

Although cold storage can markedly impair insect physiology (35, 36), cold acclimation represents an effective strategy to alleviate these detrimental effects (37). This approach has been shown to preserve the fitness of natural enemies, including *T. brassicae* (17), *O. similis* (18), and *Neoseiulus bicaudus* (38). In the present study, the survival rate of *A. custos* during cold storage was significantly lower than that observed at other stages, although it was comparable to that reported for cold-acclimated *O. similis* (18). However, survival during cold storage was slightly lower than values reported previously (12, 39), which may reflect differences in insect populations, rearing diet, or storage density (12, 40).

Because metabolites directly reflect physiological adjustments associated with cold tolerance, we used metabolomic profiling to investigate metabolic regulation in *A. custos* during cold storage. Lipids and lipid-like molecules, organic acids and derivatives, and organoheterocyclic compounds were identified as the predominant metabolite classes. The metabolic profile of *A. custos* exhibited dynamic shifts across successive stages of gradually decreasing temperature. In total, 1,275 DEMs were identified. In the LP *vs*. NP comparison, most DEMs were upregulated, suggesting that *A. custos* initiates metabolite accumulation in response to the initial temperature decline. In contrast, comparisons involving the CP stage (CP *vs*. NP and CP *vs*. LP) revealed a larger number of DEMs with predominant downregulation, indicating extensive metabolic reprogramming under prolonged cold exposure and likely increased metabolite consumption to sustain physiological homeostasis. This pattern is consistent with the reduced survival observed during the CP stage. Through integrated analysis, 46 core metabolites and eight key pathways were identified as potentially central to the cold stress response in *A. custos*. These pathways were primarily associated with lipid metabolism (e.g., glycerophospholipid metabolism and the sphingolipid signaling pathway) and carbohydrate metabolism (e.g., starch and sucrose metabolism and central carbon metabolism). Therefore, our subsequent detailed analysis focused on the regulatory mechanisms of DEMs within these two essential pathways. Notably, the metabolic shifts observed here resemble profiles reported during reproductive diapause in *A. custos* (11) and are consistent with cold-responsive strategies in *C. tujafilina* (20), *Gromphadorhina coquereliana* (41), and *Liriomyza trifolii* (42). In support of this hypothesis, no oviposition was observed in adults of *A. custos* during this study, suggesting they may have entered a state of quiescence or diapause as previously described (11); however, further studies are needed for verification.

As a critical driver of this cold adaptation and potential diapause, lipid metabolism constitutes a central component of cellular physiology, supporting membrane biogenesis, energy balance, and signaling processes. In insects, lipid remodeling is particularly important during developmental transitions and environmental stress responses (43). Under cold stress, four major lipid metabolic pathways appear to form a coordinated regulatory network that facilitates cold adaptation in *A. custos*. A key feature of this network is the maintenance of membrane fluidity and structural stability at low temperature. The pronounced accumulation of long-chain polyunsaturated phospholipids (e.g., PC (18:1/18:3), PE (20:3/18:1), PE (18:2/18:3), and PS (18:4/22:6)) during the CP stage is speculated to lower the lipid phase transition temperature. Functionally, this metabolic shift implies a robust regulatory response, likely driven by the upregulation of fatty acid desaturases and elongases. These enzymes are canonical regulators of homeoviscous adaptation in insects, actively introducing double bonds into fatty acyl chains to prevent membrane transition into a rigid gel phase (44–46). PC serves as a principal membrane phospholipid, whereas LysoPC functions as an active molecule at the biomembrane surface. The observed upregulation of long-chain LysoPC (18:1/18:3), which may promote PC synthesis via acylation, together with the downregulation of LysoPC (14:1), which may reduce phospholipase A2 (PLA2)-mediated hydrolysis, suggests targeted membrane remodeling. This continuous lipid turnover is presumably regulated by a delicate balance between PLA2 and lysophosphatidylcholine acyltransferases (47), ensuring that membrane integrity is maintained without excessive lipid degradation. We hypothesize that this strategy parallels mechanisms described in *Drosophila*, where membrane stability is maintained through precise phospholipid adjustments (48). Concurrent downregulation of PA, short-chain DGs, and CDP-DG further implies a metabolic shift favoring sustained synthesis of downstream membrane phospholipids, such as phosphatidylinositol (PI) and PS (49). Beyond structural remodeling, specific polyunsaturated DG subtypes may also exert antioxidant effects by activating cannabinoid receptor 1 (CB1), thereby inhibiting reactive oxygen species (ROS) production, and stimulating the protein kinase C epsilon (PKCϵ) pathway to enhance antioxidant enzyme activity, including glutathione peroxidase (50, 51). Within sphingolipid metabolism, overall ceramide downregulation may limit excessive apoptosis through inhibition of the mitochondrial caspase-3 pathway (52), whereas sphingomyelin (SM) accumulation may stabilize lipid rafts and preserve membrane protein function (53). This dynamic shift from ceramides to SM suggests an adaptive regulatory mechanism, likely mediated by the activation of sphingomyelin synthase (SMS) and the suppression of ceramidase, acting as a crucial survival signal under prolonged stress.

Carbohydrate metabolism is also widely recognized as a key regulator of cold stress responses in insects, with trehalose functioning as a critical “cryoprotective buffer” for osmotic balance and energy supply. Such adjustments have been documented in *A. custos*, *Bactrocera dorsalis*, and *Telenomus remus* (12, 54, 55). In the present study, carbohydrate metabolic pathways were significantly reprogrammed during cold exposure. Trehalase catalyzes the hydrolysis of trehalose into glucose, supplying substrates for glycolysis and the tricarboxylic acid (TCA) cycle (56). Trehalase activity was significantly elevated during the CP stage, consistent with previous findings in cold-stored *A. custos* (12). The regulation of trehalase and subsequent carbohydrate mobilization in insects is typically under strict neuroendocrine control, primarily governed by the adipokinetic hormone (AKH) and insulin/insulin-like growth factor signaling (IIS) pathways (57, 58). The activation of trehalase suggests that the AKH pathway may be actively stimulated during the initial phases of cold stress to liberate energy for synthesizing downstream cryoprotectants. However, our metabolomic profiling revealed no significant changes in trehalose content. This indicates a state of dynamic equilibrium driven by rapid metabolic turnover, enabling *A. custos* to continuously mobilize glucose for energy metabolism and stress adaptation without compromising its core trehalose reserves. To fuel this rapid turnover, it is also possible that the insect mobilizes alternative carbohydrates to maintain hemolymph trehalose homeostasis (59); the underlying mechanism warrants further investigation. During the CP and RP stages, significant accumulation of L-proline—a compatible solute with both osmoregulatory and antioxidant functions—may mitigate freezing injury by reducing freezable water content and stabilizing osmotic balance (60). The targeted accumulation of L-proline, alongside variations in L-aspartic acid and L-serine, suggests a potential functional reprogramming of the TCA cycle intermediates, redirecting carbon flux from energy generation toward the synthesis of molecular chaperones and antioxidants. These metabolites are also interconnected with the glutathione pathway, a central component of antioxidant defense and ROS signaling. Previous work demonstrated that GSH levels and the GSH/GSSG ratio are significantly reduced during reproductive diapause in *A. custos* (11). Additionally, marked fluctuations in adenosine monophosphate (AMP) and diacylglycerols (DGs) within the cAMP–PKA signaling pathway further imply that signal-mediated membrane remodeling and osmotic regulation may be critical for maintaining cellular homeostasis under cold stress (61).

Based on the observed metabolic remodeling, we hypothesize that the adaptation of *A. custos* to cold storage is not a passive metabolic suppression, but an active, energy-intensive process. This process is governed by a trade-off between lipid-driven membrane remodeling and the osmotic and antioxidant buffering achieved through carbohydrate and amino acid mobilization. To alleviate the energy burden of this trade-off, these insights provide a targeted framework for optimizing mass-rearing and cold storage protocols. Nutritionally, pre-storage dietary conditioning with specific precursors (e.g., L-proline) can proactively build endogenous reserves, enhancing cold hardiness without draining intrinsic energy. However, it should be noted that the mechanistic insights proposed herein rely primarily on indirect functional inferences from untargeted metabolomics. This lack of direct experimental validation remains a key limitation of the current study. Therefore, future studies are required to verify the abundance changes and elucidate exact mechanistic roles of these specific metabolites in regulating *A. custos* cold hardiness. Concurrently, future efforts should also focus on optimizing cold storage protocols—such as prolonging the cold acclimation period and implementing a fluctuating thermal regime—to facilitate energy metabolism and ROS clearance. These will significantly extend the shelf-life and preserve the biocontrol efficacy of *A. custos*. While these strategies are promising, a critical methodological consideration remains when interpreting the metabolic trajectories across the NP, LP, CP, and RP phases: the potential confounding effect of chronological aging. To minimize this bias, we adopted the methodology of Zhang et al. (11) by using adult *A. custos* at a stable developmental stage, thereby excluding major developmental shifts. Additionally, the insects may enter a state of cold-induced quiescence or diapause during cold storage. Given that temperatures below developmental thresholds severely depress metabolic rates and effectively halt physiological development and aging (62, 63), the dramatic metabolic reprogramming observed here primarily reflects targeted cold acclimation rather than routine developmental progression. Nevertheless, as subtle age-related metabolic background noise cannot be entirely ruled out, future studies employing additional physiological indicators or finer-scale experimental treatments are needed to fully elucidate the metabolic responses of *A. custos*.

## 5 Conclusions

This study provides insights into the metabolic coping strategies employed by *A. custos* during low-temperature storage. Although prolonged cold exposure reduced survival and suppressed overall metabolic activity, the insects displayed a coordinated adaptive response that appears to be primarily mediated by lipid and carbohydrate regulation. Membrane fluidity and structural integrity are hypothesized to be preserved through the accumulation of unsaturated phospholipids and sphingomyelins, together with the suppression of pro-apoptotic ceramides. At the same time, increased trehalase activity and the accumulation of compatible solutes likely functioned as essential cryoprotectants and energy sources. While additional studies are needed to further elucidate the detailed regulatory networks underlying these pathways, the present findings establish a robust theoretical basis for optimizing cold storage protocols, thereby improving the shelf-life and practical efficacy of this biocontrol agent.

## Data Availability

The data presented in the study are deposited in the OMIX database of the National Genomics Data Center (NGDC), accession number OMIX015879 (https://ngdc.cncb.ac.cn/omix/release/OMIX015879).

## Supplementary material

Supplementary Figure 1 Overlapping total ion chromatograms of the quality control samples.

Supplementary Figure 2 HMDB-based classification of differentially expressed metabolites.

Supplementary Figure 3 Trend profiles of differentially expressed metabolites involved in lipid and carbohydrate metabolism pathways.

Supplementary Table 1 Top 20 KEGG pathway classifications of differentially expressed metabolites.

## References

[1] Zhao Q Wei J Bu W Liu G Zhang H . Synonymize Arma chinensis as Arma custos based on morphological, molecular and geographical data. Zootaxa. (2018) 4455:161–76. doi: 10.11646/zootaxa.4455.1.7 30314225

[2] Zou DY Wang MQ Zhang LS Zhang Y Chen HY . Taxonomic and bionomic notes on Arma chinensis (Fallou) (Hemiptera: Pentatomidae: Asopinae). Zootaxa. (2012) 3382:41–52. doi: 10.11646/zootaxa.3382.1.4

[3] Wang YQ Luo YF Ge YK Liu S Liang WK Wu CY . Chromosome-level genome assembly of the predatory stink bug Arma custos. Sci Data. (2024) 11:417. doi: 10.1038/s41597-024-03270-8 38654007 PMC11039643

[4] Rider DA Zheng L Kerzhner IM . Checklist and nomenclatural notes on the Chinese Pentatomidae (Heteroptera). II. Pentatominae. Zoosyst Ross. (2002) 11:135–53. doi: 10.31610/zsr/2002.11.1.135 41770980

[5] Pan M Zhang H Zhang L Chen H . Effects of starvation and prey availability on predation and dispersal of an omnivorous predator Arma chinensis Fallou. J Insect Behav. (2019) 32:134–44. doi: 10.1007/s10905-019-09718-9 30311153

[6] Liu J Liu X Liao J Li C . Biological performance of Arma chinensis on three preys Antheraea pernyi, Plodia interpunctella and Leptinotarsa decemlineata. Int J Pest Manag. (2025) 71:377–84. doi: 10.1080/09670874.2023.2216173 37339054

[7] Tang YT Li YY Liu CX Mao JJ Chen HY Zhang LS . Predation and behavior of Arma chinensis to Spodoptera frugiperda. Plant Prot. (2019) 45:65–8. doi: 10.16688/j.zwbh.2019264

[8] Tang YT Guo Y Pan MZ Mao JJ Chen HY Zhang LS . Predation of Plutella xylostella larva by Arma chinensis. Plant Prot. (2020) 46:155–60. doi: 10.16688/j.zwbh.2019243

[9] Liu J Liao JH Li C . Functional response and control potential of adult Arma chinensis on Colorado potato beetle in Xinjiang, China. CABI Agric Biosci. (2024) 5:100. doi: 10.1186/s43170-024-00306-2 38164791

[10] Pan MZ Fu ZX Li YY Chen HY Zhang LS Liu TX . Role of host plants in the suitability and dispersal of an omnivorous predator Arma chinensis Fallou (Hemiptera: Pentatomidae: Asopinae) in a biological control context. J Plant Dis Prot. (2022) 129:861–8. doi: 10.1007/s41348-022-00624-5 30311153

[11] Zhang MS He WW Li YY Chen JJ Teets NM Zhang LS . Metabolic and transcriptional regulation of reproductive diapause in Arma chinensis. iScience. (2025) 28:111761. doi: 10.1016/j.isci.2025.111761 40124477 PMC11928864

[12] Guo Y Zhang J Li PY Li DS . Effect of low-temperature storage on survival rate and major biochemical substances of Arma chinensis adult. Chin J Biol Control. (2025) 41:1241–8. doi: 10.16409/j.cnki.2095-039x.2024.11.011

[13] Colinet H Boivin G . Insect parasitoids cold storage: a comprehensive review of factors of variability and consequences. Biol Control. (2011) 58:83–95. doi: 10.1016/j.biocontrol.2011.04.014 38826717

[14] Wang RZ Zhang Y Wang XD Song LW Li H Liu XR . Research progress on cold storage tolerance of natural enemy insects. Chin J Biol Control. (2021) 43:1408–16. doi: 10.3969/j.issn.1674-0858.2021.06.08

[15] Hance T van Baaren J Vernon P Boivin G . Impact of extreme temperatures on parasitoids in a climate change perspective. Annu Rev Entomol. (2007) 52:107–26. doi: 10.1146/annurev.ento.52.110405.091333 16846383

[16] Xia PL Wang B Xie XF Feng Y Huang Y . Effect of temperature on survival and immature development of Arma chinensis. J Asia-Pac Entomol. (2022) 25:101927. doi: 10.1016/j.aspen.2022.101927 38826717

[17] Lessard E Boivin G . Effect of low temperature on emergence, fecundity, longevity and host-feeding by Trichogramma brassicae. Biocontrol. (2013) 58:319–29. doi: 10.1007/s10526-012-9493-8 30311153

[18] Zeng G Zhi JR Zhang CR Mao Y Tao Z . Effects of cold acclimation on cold storage of Orius similis. Chin J Biol Control. (2019) 35:20–3. doi: 10.16409/j.cnki.2095-039x.2019.01.006

[19] Koštál V Korbelová J Rozsypal J Zahradníčková H Cimlová J Tomčala A . Long-term cold acclimation extends survival time at 0 °C and modifies the metabolomic profiles of the larvae of the fruit fly Drosophila melanogaster. PloS One. (2011) 6:e25025. doi: 10.1371/journal.pone.0025025 21957472 PMC3177886

[20] Durak R Durak T . Metabolic response of aphid Cinara tujafilina to cold stress. Biology. (2021) 10:1288. doi: 10.3390/biology10121288 34943203 PMC8698524

[21] Mojib-Haghghadam Z Sendi JJ Zibaee A Mohaghegh J . Effect of cold storage on some biological and physiological performance of Adalia decempunctata L. Comp Biochem Physiol B. (2023) 263:110797. doi: 10.1016/j.cbpb.2022.110797 36064137

[22] Khabir M Izadi H Mahdian K . The supercooling point depression is the leading cold tolerance strategy for the variegated ladybug, Hippodamia variegata (Goezel). Front Physiol. (2023) 14:1323701. doi: 10.3389/fphys.2023.1323701 38179144 PMC10764430

[23] Li XP . Influence of two artificial diets on development and cold storage of Arma custos (Hemiptera: Pentatomidae). Beijing: Beijing Forestry University (2020). dissertation.

[24] Cagnotti CL Lois M López SN Botto EN Viscarret MM . Cold storage of Trichogramma nerudai using an acclimation period. Biocontrol. (2018) 63:565–73. doi: 10.1007/s10526-018-9885-5 30311153

[25] Wei L Yang MF Huang N Ou HD Wang XQ Huang Y . Effects of cold storage after cold acclimation on the fitness of Habrobracon hebetor (Hymenoptera: Braconidae). J Econ Entomol. (2023) 116:1496–504. doi: 10.1093/jee/toad134 37476852

[26] Zhong Y Liao X Hou M . Predatory capacity and reproduction of Cyrtorhinus lividipennis (Hemiptera: Miridae) adults exposed to low-temperature storage and fitness of the F1 generation. Insects. (2023) 14:226. doi: 10.3390/insects14030226 36975911 PMC10054917

[27] Pan CN Zhou W Lu CH Pan YN Liu LY Chen WL . Fitness implications of low-temperature storage for Eocanthecona furcellata (Hemiptera: Pentatomidae). J Econ Entomol. (2024) 117:1739–52. doi: 10.1093/jee/toae199 39241700

[28] Jensen K Michaelsen JV Larsen MT Kristensen TN Holmstrup M Overgaard J . Increased lipid accumulation but not reduced metabolism explains improved starvation tolerance in cold-acclimated arthropod predators. Sci Nat. (2018) 105:65. doi: 10.1007/s00114-018-1593-6 30456565

[29] Leon-Quinto T Serna A . Cryoprotective response as part of the adaptive strategy of the red palm weevil, Rhynchophorus ferrugineus, against low temperatures. Insects. (2022) 13:134. doi: 10.3390/insects13020134 35206708 PMC8879650

[30] Guo Y. Li D. S. Zhao C. Li J. Z. Song Z. W. (2022). A method for cold storage of Arma chinensis adults. China Patent No. 202010201120.3. China National Intellectual Property Administration.

[31] Li C Al-Dalali S Zhou H Xu B . Influence of curing on the metabolite profile of water-boiled salted duck. Food Chem. (2022) 397:133752. doi: 10.1016/j.foodchem.2022.133752 35917791

[32] Xu Y Diao L Yang X Zhao M Xi Y Liu Y . Changes in metabolomics profiles of Propylea japonica in response to acute heat stress. Int J Mol Sci. (2025) 26:4541. doi: 10.3390/ijms26104541 40429686 PMC12110978

[33] Gupta R Laxman S . Steady-state and flux-based trehalose estimation as an indicator of carbon flow from gluconeogenesis or glycolysis. Bio-protocol. (2020) 10:e3483. doi: 10.21769/BioProtoc.3483 32181267 PMC7075708

[34] Chang Y Zhang B Geng Z Wei J An S Zhao W . Molecular mechanism of azadirachtin inhibiting trehalase activity leading to abnormal molting and metamorphosis of Helicoverpa armigera larvae. Chin J Biol Control. (2023) 39:550–9. doi: 10.16409/j.cnki.2095-039x.2022.03.015

[35] Rebaudo F Rabhi V . Modeling temperature-dependent development rate and phenology in insects: review of major developments, challenges, and future directions. Entomol Exp Appl. (2018) 166:607–17. doi: 10.1111/eea.12693 40046247

[36] Rismayani UMS Chi H Gotoh T . Impact of constant and fluctuating temperatures on population characteristics of Tetranychus pacificus (Acari: Tetranychidae). J Econ Entomol. (2021) 114:638–51. doi: 10.1093/jee/toaa327 33547783

[37] Xie Z Xu L Zhao J Li N Qin D Xiao C . Rapid cold hardening and cold acclimation promote cold tolerance of oriental fruit fly, Bactrocera dorsalis (Hendel) by physiological substances transformation and cryoprotectants accumulation. Bull Entomol Res. (2023) 113:574–86. doi: 10.1017/s0007485323000251 37501573

[38] Tang SQ Zuo L Su J Cai ZP Zhang JP . Cold acclimation enhances cold tolerance and storage capacity in Neoseiulus bicaudus (Acari: Phytoseiidae). J Econ Entomol. (2025) 118:2749–59. doi: 10.1093/jee/toaf262 41071916

[39] Li XP Song LW Coudron TA Zuo TT Chen YQ Zhang Y . Effects of two natural diets on the response of the predator Arma chinensis (Hemiptera: Pentatomidae: Asopinae) to cold storage. Appl Ecol Environ Res. (2019) 17:15329–47. doi: 10.15666/aeer/1706_1532915347

[40] Pourani MS Mahdian K Izadi H Basirat M Sahhafi SR . Cold tolerance and supercooling points of two ladybird beetles (Col.: Coccinellidae): impact of the diet. Cryobiology. (2019) 91:61–8. doi: 10.1016/j.cryobiol.2019.10.197 31669223

[41] Chowański S Lubawy J Spochacz M Paluch E Smykalla G Rosinski G . Cold induced changes in lipid, protein and carbohydrate levels in the tropical insect Gromphadorhina coquereliana. Comp Biochem Phys A. (2015) 183:57–63. doi: 10.1016/j.cbpa.2015.01.007 25624163

[42] Zhang XX Iqbal J Wang YC Chang YW Hu J Du YZ . Integrated transcriptional and biochemical profiling suggests mechanisms associated with rapid cold hardening in adult Liriomyza trifolii (Burgess). Sci Rep. (2024) 14:24033. doi: 10.1038/s41598-024-75146-1 39402107 PMC11473728

[43] Arrese EL Soulages JL . Insect fat body: energy, metabolism, and regulation. Annu Rev Entomol. (2010) 55:207–25. doi: 10.1146/annurev-ento-112408-085356 19725772 PMC3075550

[44] Wu G Baumeister R Heimbucher T . Molecular mechanisms of lipid-based metabolic adaptation strategies in response to cold. Cells. (2023) 12:22. doi: 10.3390/CELLS12101353 37408188 PMC10216534

[45] Ernst R Ejsing CS Antonny B . Homeoviscous adaptation and the regulation of membrane lipids. J Mol Biol. (2016) 428:4776–91. doi: 10.1016/j.jmb.2016.08.013 27534816

[46] Ullah F Abbas A Gul H Güncan A Hafeez M Gadratagi BG . Insect resilience: unraveling responses and adaptations to cold temperatures. J Pest Sci. (2024) 97:1153–69. doi: 10.1007/s10340-023-01741-2 30311153

[47] Wattelet-Boyer V Guédard ML Dittrich-Domergue F Maneta-Peyret L Kriechbaumer V Boutté Y . Lysophosphatidic acid acyltransferases: a link with intracellular protein trafficking in Arabidopsis root cells? J Exp Bot. (2022) 73:1327–43. doi: 10.1093/jxb/erab504 34982825

[48] Moos M Korbelová J Štětina T Opekar S Šimek P Grgac R . Cryoprotective metabolites are sourced from both external diet and internal macromolecular reserves during metabolic reprogramming for freeze tolerance in drosophilid fly, Chymomyza costata. Metabolites. (2022) 12:163. doi: 10.3390/metabo12020163 35208237 PMC8877510

[49] Lehmann M . Diverse roles of phosphatidate phosphatases in insect development and metabolism. Insect Biochem Mol Biol. (2020) 133:103469. doi: 10.1016/j.ibmb.2020.103469 32931938 PMC7952469

[50] Chen CH Pan J Di YQ Liu W Hou L Wang JX . Protein kinase C delta phosphorylates ecdysone receptor B1 to promote gene expression and apoptosis under 20-hydroxyecdysone regulation. Proc Natl Acad Sci. (2017) 114:E7121–30. doi: 10.1073/pnas.1704999114 28790182 PMC5576805

[51] Palomba L Silvestri C Imperatore R Morello G Di Marzo V . Negative regulation of leptin-induced reactive oxygen species (ROS) formation by CB1 receptor activation in hypothalamic neurons. J Biol Chem. (2015) 290:13669–81. doi: 10.1074/jbc.M115.646885 25869131 PMC4447947

[52] Hannun YA Obeid LM . Principles of bioactive lipid signalling: lessons from sphingolipids. Nat Rev Mol Cell Biol. (2008) 9:139–50. doi: 10.1111/j.1540-8159.2005.00043.x 18216770

[53] Simons K Ikonen E . Functional rafts in cell membranes. Nature. (1997) 387:569–72. doi: 10.1038/42408 9177342

[54] Yu C Zhao R Zhou W Pan Y Tian H Yin Z . Fruit fly in a challenging environment: impact of short-term temperature stress on the survival, development, reproduction, and trehalose metabolism of Bactrocera dorsalis (Diptera: Tephritidae). Insects. (2022) 13:753. doi: 10.3390/insects13080753 36005378 PMC9410078

[55] Chen WB Liu H Li YY Wang MQ Mao JJ Guo ZJ . Trehalose accumulation contributes to enhanced cold stress tolerance in Telenomus remus, a dominant egg parasitoid of Spodoptera frugiperda. BMC Genomics. (2025) 26:825. doi: 10.1186/s12864-025-12059-x 41013228 PMC12465949

[56] Tellis MB Kotkar HM Joshi RS . Regulation of trehalose metabolism in insects: from genes to the metabolite window. Glycobiology. (2023) 33:262–73. doi: 10.1093/glycob/cwad011 36762907

[57] Lu K Wang Y Chen X Zhang X Li W Cheng Y . Adipokinetic hormone receptor mediates trehalose homeostasis to promote vitellogenin uptake by oocytes in Nilaparvata lugens. Front Physiol. (2019) 9:1904. doi: 10.3389/fphys.2018.01904 30687120 PMC6338042

[58] Baki MAA Jung JK Kim Y . Regulation of hemolymph trehalose titers by insulin signaling in the legume pod borer, Maruca vitrata (Lepidoptera: Crambidae). Peptides. (2018) 106:28–36. doi: 10.1016/j.peptides.2018.06.006 29935203

[59] Zhang DW Xiao ZJ Zeng BP Li K Tang YL . Insect behavior and physiological adaptation mechanisms under starvation stress. Front Physiol. (2019) 10:163. doi: 10.3389/fphys.2019.00163 30890949 PMC6411660

[60] Koštál V Korbelová J Poupardin R Moos M Šimek P . Arginine and proline applied as food additives stimulate high freeze tolerance in larvae of Drosophila melanogaster. J Exp Biol. (2016) 219:2358–67. doi: 10.1242/jeb.142158 27489218

[61] Evans JJ Xiao CF Robertson RM . AMP-activated protein kinase protects against anoxia in Drosophila melanogaster. Comp Biochem Physiol A. (2017) 214:30–9. doi: 10.1016/j.cbpa.2017.09.006 28916374

[62] Denlinger DL Lee RE . Low temperature biology of insects. Denlinger DL Lee RE , editors. Cambridge: Cambridge University Press (2010). doi: 10.1017/CBO9780511675997

[63] Stejskal V Vendl T Li Z Aulicky R . Minimal thermal requirements for development and activity of stored product and food industry pests (Acari, Coleoptera, Lepidoptera, Psocoptera, Diptera and Blattodea): a review. Insects. (2019) 10:149. doi: 10.3390/insects10050149 31126156 PMC6571962

