# Supplementary material for: Comparative metabolomics provides insights into the metabolic reprogramming of the predatory Arma custos (Fabricius) during low-temperature storage
Source: Front Insect Sci. 2026 Jun 5;6:1820584. doi: 10.3389/finsc.2026.1820584 (PMC13279525; doi:10.3389/finsc.2026.1820584)
Supplement: Supplementary Figure 1 — Overlapping total ion chromatograms of the quality control samples. [file Supplementaryfile1.docx]

Supplementary Material


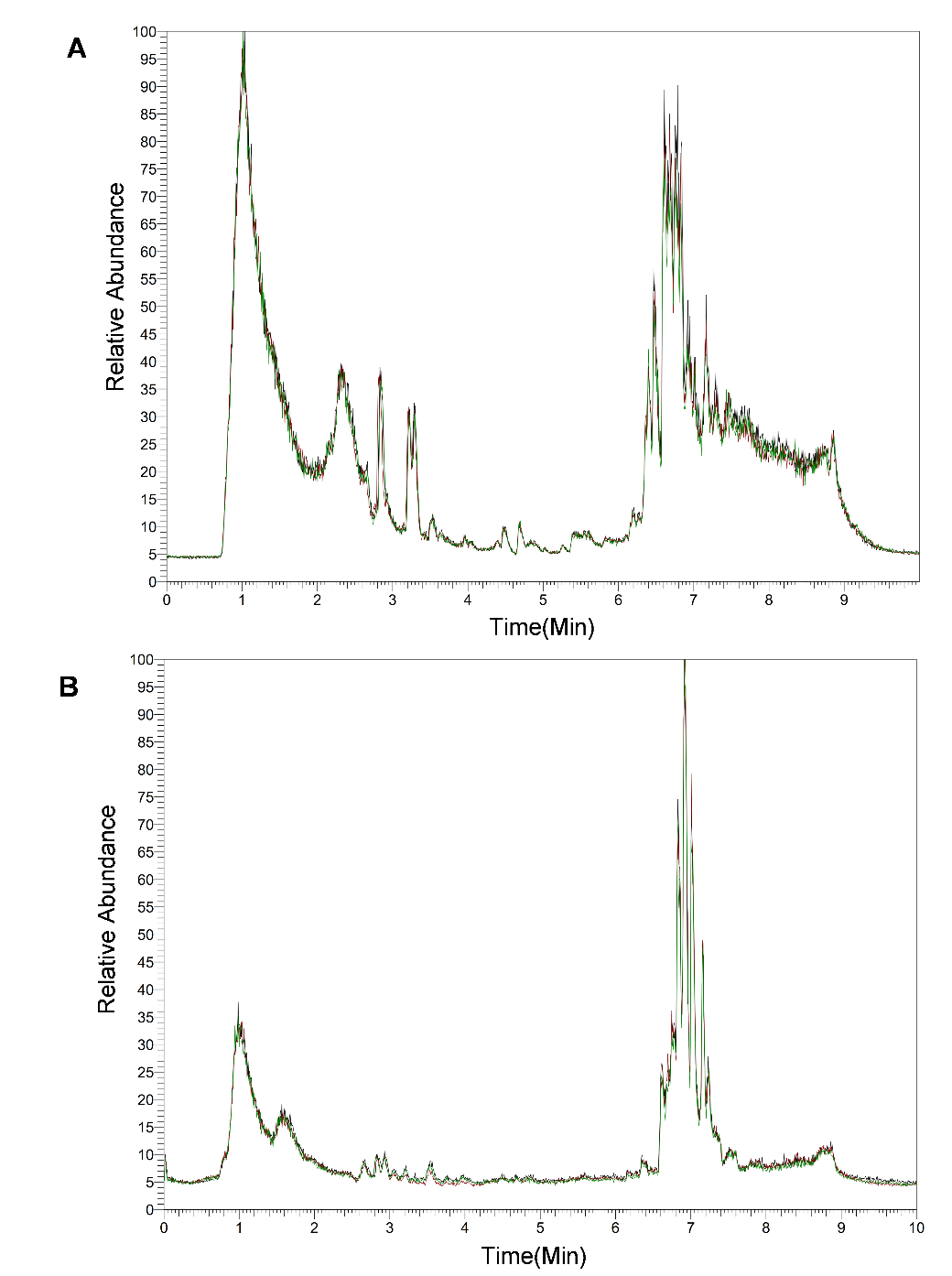


**Supplementary Figure 1.** Overlapping total ion chromatograms of the quality control samples.


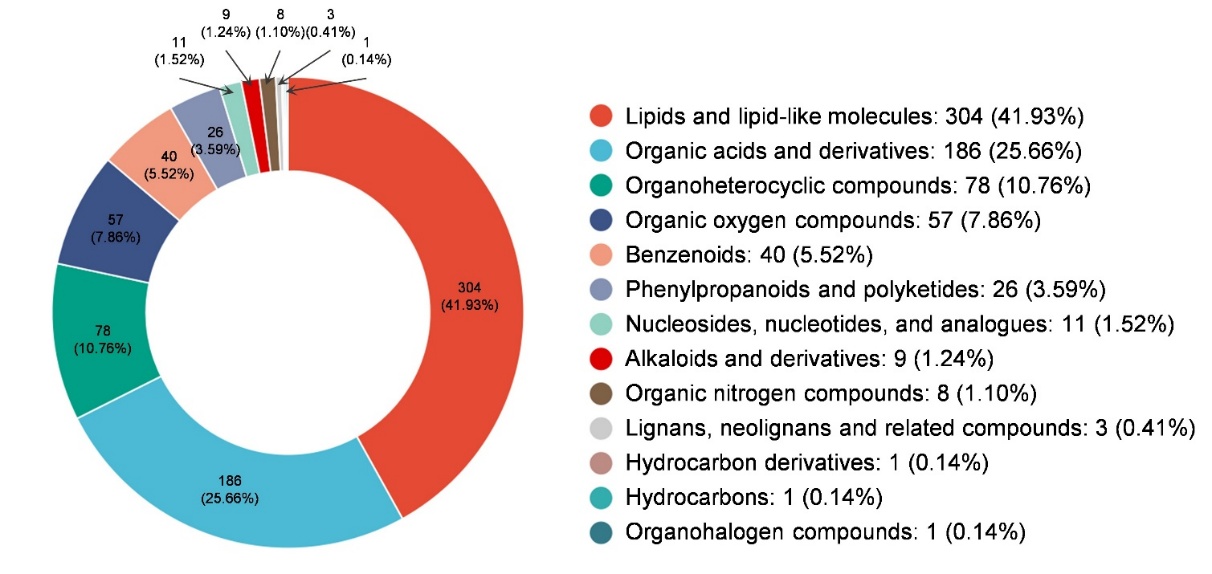


**Supplementary Figure 2.** HMDB-based classification of differentially expressed metabolites.


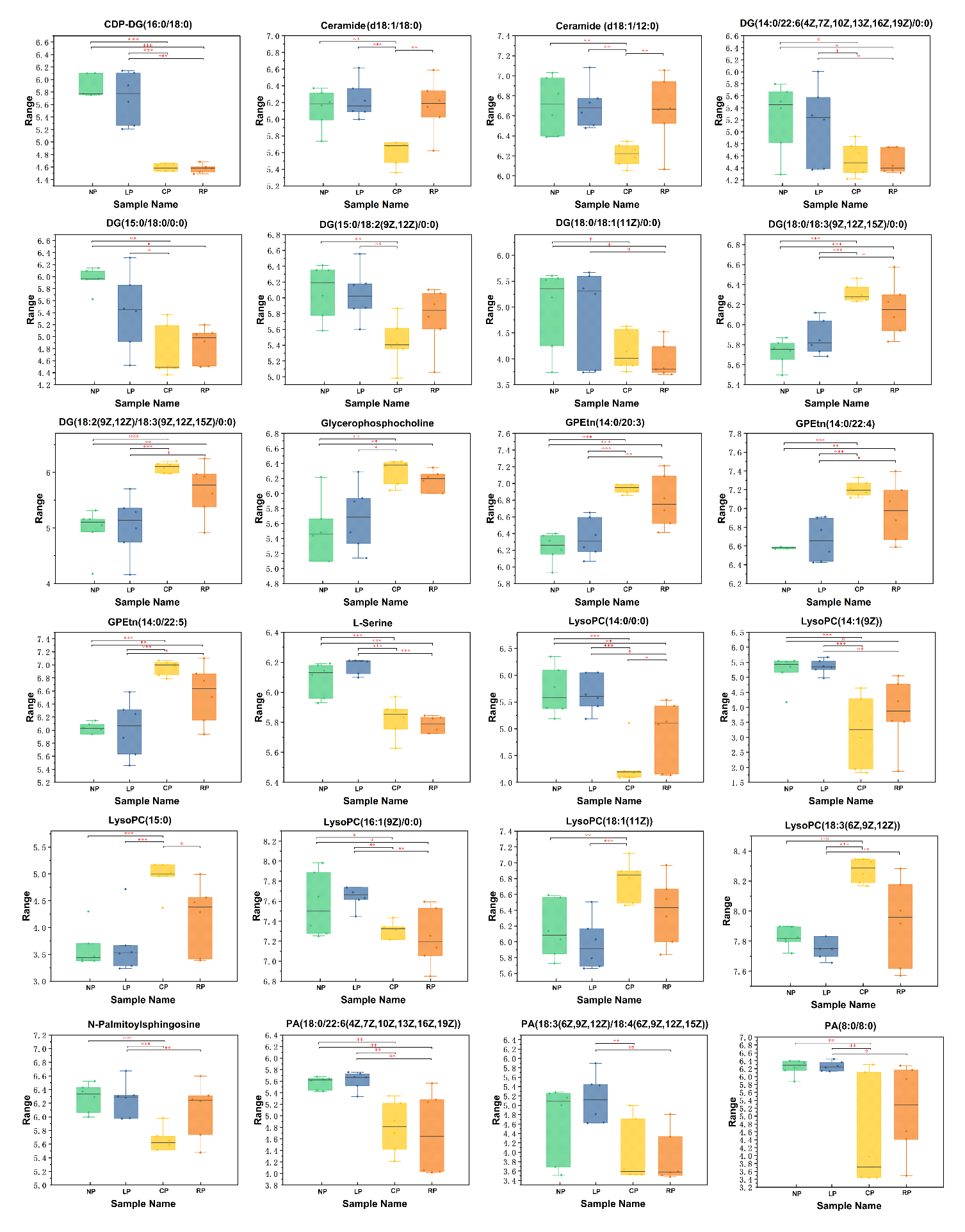

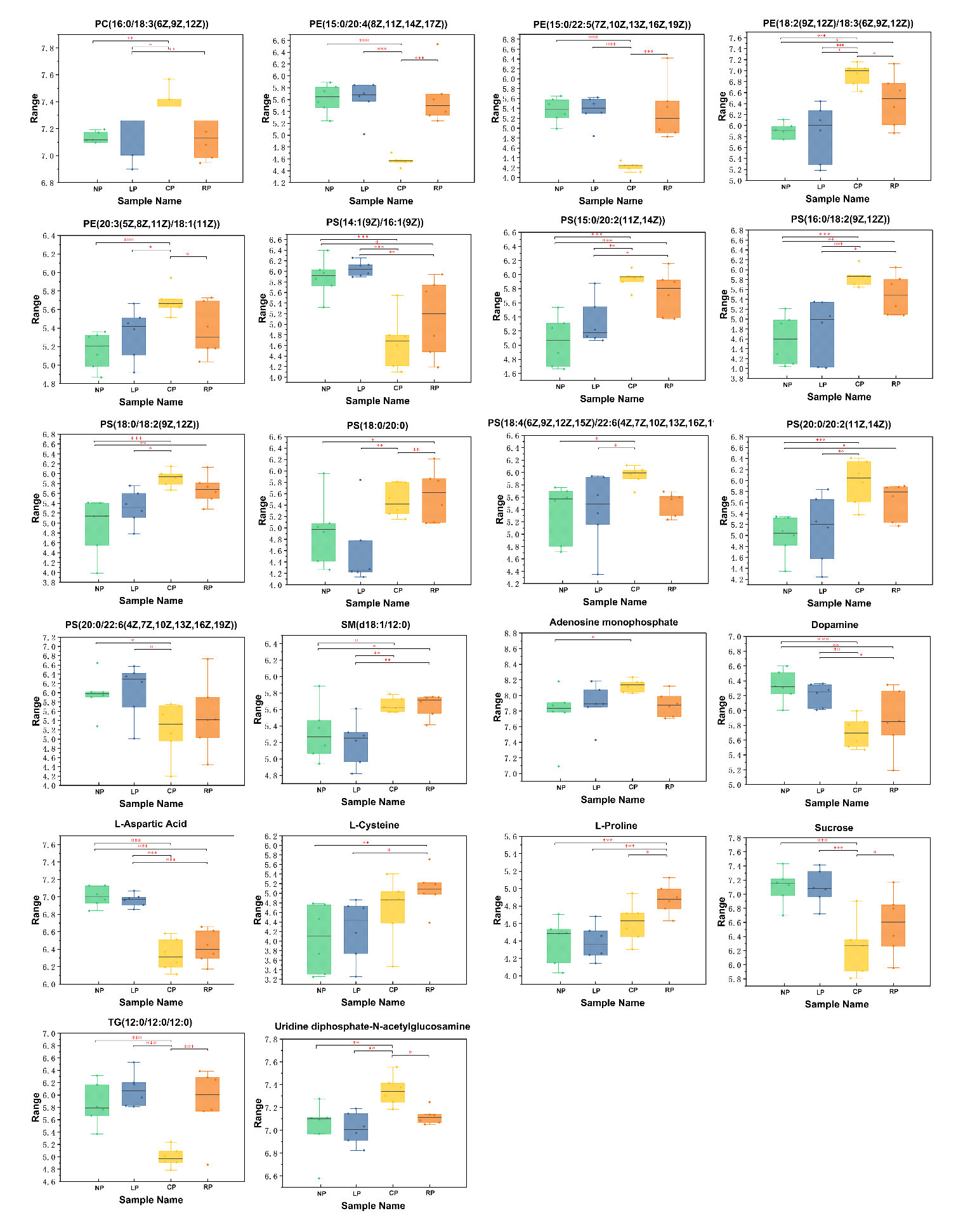


**Supplementary Figure 3.** Trend profiles of differentially expressed metabolites involved in lipid and carbohydrate metabolism pathways.

**Supplementary Table 1.** Top 20 KEGG pathway classifications of differentially expressed metabolites.

| **Metabolic Pathway** | **Total** | **Percentage (%)** | **KEGG Classification** |
| --- | --- | --- | --- |
| Lipid metabolism | 54 | 14.59% | Metabolism |
| Amino acid metabolism | 46 | 12.43% | Metabolism |
| Cancer: overview | 30 | 8.11% | Human Diseases |
| Nervous system | 25 | 6.76% | Organismal Systems |
| Digestive system | 23 | 6.22% | Organismal Systems |
| Endocrine system | 21 | 5.68% | Organismal Systems |
| Infectious disease: parasitic | 18 | 4.86% | Human Diseases |
| Signal transduction | 18 | 4.86% | Environmental Information Processing |
| Infectious disease: viral | 15 | 4.05% | Human Diseases |
| Infectious disease: bacterial | 14 | 3.78% | Human Diseases |
| Metabolism of cofactors and vitamins | 14 | 3.78% | Metabolism |
| Cardiovascular disease | 13 | 3.51% | Human Diseases |
| Endocrine and metabolic disease | 12 | 3.24% | Human Diseases |
| Nucleotide metabolism | 11 | 2.97% | Metabolism |
| Carbohydrate metabolism | 10 | 2.70% | Metabolism |
| Immune system | 10 | 2.70% | Organismal Systems |
| Cancer: specific types | 9 | 2.43% | Human Diseases |
| Drug resistance: antineoplastic | 9 | 2.43% | Human Diseases |
| Immune disease | 9 | 2.43% | Human Diseases |
| Membrane transport | 9 | 2.43% | Environmental Information Processing |
